# Successful Management of Pseudo-Ludwig Angina from Supratherapeutic Warfarin Use: A Case Report

**DOI:** 10.5811/cpcem.20386

**Published:** 2025-01-19

**Authors:** Utku Ekin, Arham Hazari, Nizar Alyassin, Alan Alcantara, Moh’d Hazem Azzam, Mourad Ismail

**Affiliations:** * St. Joseph’s University Medical Center, Department of Pulmonary and Critical Care Medicine, Paterson, New Jersey; † St. Joseph’s University Medical Center, Department of Internal Medicine, Paterson, New Jersey

**Keywords:** pseudo-Ludwig angina, warfarin, anticoagulation, sublingual hematoma, case report

## Abstract

**Introduction:**

Ludwig angina is a potentially fatal condition characterized by soft tissue infection of the submandibular, sublingual, and submental compartments. Pseudo-Ludwig angina is a rare condition characterized by sublingual swelling of non-infectious etiology, typically in the setting of supratherapeutic anticoagulation. However, other etiologies, such as angioedema and trauma, have been described.

**Case Report:**

We present the case of a 59-year-old female with pseudo-Ludwig angina that developed in the setting of warfarin therapy and supratherapeutic international normalized ratio. She presented with sublingual swelling and dysphagia. She was successfully treated with dexamethasone, vitamin K, and fresh frozen plasma. The most appropriate imaging modalities in these cases are contrast-enhanced computed tomography (CT) and CT angiogram. If a hematoma is present, antibiotics for anaerobic coverage are also appropriate.

**Conclusion:**

We hope this case sheds light upon this rare pathology and ultimately hastens recognition and proper intervention.

## INTRODUCTION

Pseudo-Ludwig angina (also known as spontaneous sublingual hematoma) is an uncommon condition in which hematoma or soft-tissue swelling arises in the sublingual compartments and may provoke significant airway obstruction.^1^ In contrast to true Ludwig angina, which is secondary to severe infection, pseudo-Ludwig angina results from non-infectious etiologies.^2^–^5^ There have been cases describing this phenomenon due to ill-fitting dentures, trauma, and even possibly uncontrolled hypertension.^6^ In 1976 Kiviranta published the first reported case of sublingual hematoma due to coagulopathy; since then the number of reported cases have been scarce.^7^ Anticoagulation with warfarin was reported in the majority of cases, although there have been cases associated with liver disease coagulopathy and direct oral anticoagulant (DOAC) therapy.^1^,^8^,^9^ It is important to note that this phenomenon has been reported for patients on anticoagulants within therapeutic range and even in patients who are not on any anticoagulants.^4^ Herein, we present a case of pseudo-Ludwig angina, which was identified and treated swiftly with conservative management, thereby preventing further airway compromise.

## CASE REPORT

**Image 1:**
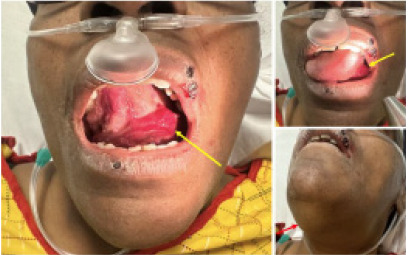
Images demonstrating the extent of tissue swelling in the sublingual space, ecchymoses (yellow arrows) clearly visible when patient was asked to raise tongue to the hard palate. Submental swelling is also prominent (red arrow).

A 59-year-old female presented to the emergency department (ED) with complaints of tongue swelling and difficulty swallowing, which she noticed when she woke up from sleep in the morning. She was in her usual state of health prior to going to sleep the night before and could not recall any inciting events or trauma to the area. She had a history of hypertension, severe mitral regurgitation, and mitral valve replacement several years prior and was taking warfarin daily. She denied any recent changes to her diet or taking any new medications. She denied taking any herbal supplements. Her warfarin dose had been recently increased from 8 milligrams (mg) daily to 11 mg daily and she had been unable to get her weekly bloodwork done in the prior two weeks. On presentation, her vitals demonstrated a blood pressure of 138/80 millimeters of mercury (mmHg), heart rate of 76 beats per minute, respiratory rate of 18 breaths per minute, and temperature of 37.1°C. She was placed on 2 liters/minute nasal cannula for comfort in the ED as she reported mild difficulty breathing. Her tongue and sublingual area appeared swollen with signs of ecchymosis. She additionally was noted to have some drooling from the right side of her mouth (Image 1).

**Image 2:**
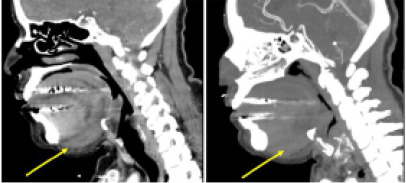
Computed tomography imaging with contrast (on the left) and angiogram (on the right) demonstrates soft-tissue swelling (yellow arrows) in the hypopharynx without any active bleeding. Images are slightly distorted due to dental artifacts

She was initially given dexamethasone 10 mg intravenous (IV) due to the swelling, and the critical care team was consulted for concern of airway compromise. Computed tomography (CT) was obtained with contrast and did not reveal any active IV contrast extravasation suggestive of active bleeding; however, there was significant swelling in the sublingual space (Image 2).

Labs revealed supratherapeutic international normalized ratio (INR) of 9.0 (reference range 0.8–1.1) as well as hemoglobin of 8.3 grams per deciliter (g/dL) (13.5–17.5 g/dL), which was low compared to her baseline hemoglobin of 11.2 g/dL. She denied any melena or hematochezia and denied any gross hematuria. As the patient was protecting her airway and reported mild improvement in symptoms, the decision was made to monitor her in the medical intensive care unit.

**CPC-EM Capsule**

What do we already know about this clinical entity?

*Pseudo-Ludwig angina (also known as spontaneous sublingual hematoma) is a rare condition that is often mistaken for Ludwig angina or angioedema*.

What makes this presentation of disease reportable?

*The patient had significant ecchymosis of the sublingual space in the setting of supratherapeutic anticoagulation*.

What is the major learning point?

*Treatment approaches to Ludwig and pseudo-Ludwig angina are different. Proper history and examination of the sublingual space is important when considering these diagnoses*.

How might this improve emergency medicine practice?

*When a patient presents with complaints of sublingual/submental swelling, keeping pseudo-Ludwig angina on the list of differentials will speed up diagnosis and treatment*.

**Image 3:**
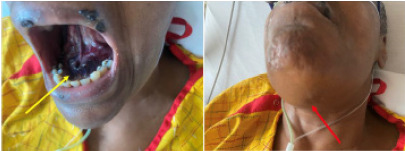
Images demonstrate the appearance of the sublingual ecchymosis (yellow arrow) and the extent of swelling (red arrow) the next day after admission.

We administered phytonadione (vitamin K_1_) 10 mg IV as well as 3 units of fresh frozen plasma to the patient to bring INR to therapeutic levels. The next morning, her INR level decreased to 1.2. She reported improvement in her breathing as well as her ability to swallow her secretions (Image 3). The sublingual swelling improved gradually, and she was discharged several days later with outpatient follow-up.

## DISCUSSION

Ludwig angina is a well-known and frequently managed diagnosis. Ludwig angina pathology includes infectious etiology that leads to cellulitis of the sublingual, submental, and submandibular compartments.^10^,^11^ Pseudo-Ludwig angina, on the other hand, is described as soft-tissue swelling in the same sublingual, submental, and/or submandibular compartments, although it is due to non-infectious causes. Non-infectious causes have been described in the literature as trauma, ill-fitted dentures, uncontrolled hypertension, supratherapeutic anticoagulation, and even angioedema.^2^–^6^,^8^,^9^ Pseudo-Ludwig angina has been reported less frequently compared to Ludwig angina and often presents in patients on anticoagulation such as warfarin or DOACs.^1^ As INR levels increase, the risk of bleeding increases, and often the suggested etiology is bleeding in the sublingual compartments that progresses to a hematoma.^8^,^12^

While Ludwig angina and pseudo-Ludwig angina presents very similarly they have different management pathways. Both entities present with swelling under the tongue that can lead to complaints of difficulty breathing and difficulty swallowing.^12^ Furthermore, on physical exam, there may be signs of sublingual ecchymosis (Image 1). Unlike Ludwig angina, pseudo-Ludwig angina has no infectious etiology, and patients do not present with fever or chills and lack leukocytosis.^2^

In our case the patient, who was taking warfarin for mechanical heart valve, presented with difficulty swallowing and sublingual ecchymosis. Although imaging did not reveal a definitive hematoma in the sublingual compartment, there was significant soft-tissue swelling. As has been suggested in previous literature, this could be due to angioedema from the anticoagulant medications as opposed to frank bleeding.^2^ In these cases steroids can help reduce swelling and inflammation. Additionally, an argument can be made to use steroid therapy even in cases of hematoma presence, as blood in tissues is known to cause local inflammation. It is, therefore, essential for clinicians to recognize these signs and symptoms due to the potential risk of misclassification and mistreatment. If imaging does not demonstrate a hematoma in this sublingual space, angioedema should be considered as a strong possibility for etiology.

The recommended imaging modality is contrast-enhanced CT. In addition, obtaining a CT angiogram may demonstrate active extravasation of contrast suggestive of active bleeding, in which case the airway should be secured with prompt endotracheal intubation. In the case of this patient, imaging demonstrated soft-tissue swelling without active contrast extravasation, as was also reported by the radiologist. No comments were made regarding the presence of a hematoma in the sublingual space. However, the CT angiogram report did describe this soft-tissue swelling as a “mass.” Previously, the presence of hematoma on CT imaging with contrast has been reported in the literature as a hyperdense lesion/mass.^3^,^4^

Many patients with mechanical heart valves are prescribed warfarin with INR goals of 2.5–3.5.^13^ Close monitoring of patients is always of great importance due to changes in diet, medication dosages, other prescribed medications, and metabolism rate leading to drastic elevation or decline of INR.^14^ In patients whose INR levels exceed 4.5 the risk of bleeding and further complications increases exponentially, and some studies have shown that the increased risk of bleeding is also noted with INR levels greater than 3.5.^15^,^16^ In this case, with an increased INR >9, the only apparent physical exam presentation was the sublingual ecchymosis and swelling. The patient was then noted at admission to have pseudo-Ludwig angina, and prompt treatment was started.

When managing both Ludwig angina and pseudo-Ludwig angina, the priority is airway management, as the enlarging sublingual compartment due to infection or hematoma can lead to airway compromise.^12^,^17^ If not recognized early, patients can deteriorate quickly with sequelae of complications such as anoxic brain injury or death. In one retrospective study, awake tracheostomy was found to be superior in safety to endotracheal intubation.^10^ Other managements include steroids to aid in inflammation and avoid the necessity for intubation or tracheostomy.^10^ Ludwig and pseudo-Ludwig angina share this management; however, pseudo-Ludwig angina does not have an infectious cause and would generally not benefit from antibiotic treatment.^2^ Although arguments have been made in cases with a hematoma, the blood collection may serve as a nidus for infection and, thus, anaerobic coverage should be initiated.^2^,^3^,^6^

Correcting the INR can be achieved by holding anticoagulant medication. Vitamin K can also be administered by IV or oral route based on the degree of INR elevation.^18^ In the retrospective Supra-War-K study, it was noted that administering vitamin K at any dose while also holding warfarin dose showed a more significant decline in INR compared to holding warfarin alone.^19^ Additionally we administered 3 units of fresh frozen plasma (FFP), and the patient’s INR the following day decreased to 1.2. She was then started on continuous heparin infusion and a low dose of warfarin 48 hours after admission to bring INR back to therapeutic levels. To reverse coagulopathy, in addition to FFP, prothrombin concentrate complex (PCC) and tranexamic acid can also be administered as initial treatment.^2^,^4^,^9^

While no clear guidelines exist for the management of pseudo-Ludwig angina, prompt recognition and treatment is of utmost importance. This can be achieved by thorough history and physical exam, looking for sublingual compartment swelling and potential signs of ecchymosis. Imaging with contrast-enhanced CT and CT angiogram is also crucial as it can reveal presence (or absence) of hematoma and whether there is active extravasation of contrast suggestive of active bleeding. In cases of coagulopathy, reversal with FFP, tranexamic acid, and PCC is appropriate. Steroids can also help control inflammation and further swelling, especially in cases of possible angioedema, although they are not frequently used in cases of obvious presence of hematoma. Antibiotics have also been used with focus on anaerobic coverage as collection of blood can serve as a nidus of infection.

We present this case in the hope of increasing awareness of pseudo-Ludwig angina in patients on oral anticoagulation. Given that many patients are now placed on anticoagulation for a variety of pathologies, recognizing pseudo-Ludwig angina will improve patient care, limit the administration of unnecessary antibiotics, and facilitate the consultation of appropriate services such as critical care and oral maxillofacial surgery.

## CONCLUSION

The management of pseudo-Ludwig angina differs from its infectious counterpart. Swift recognition and correction of the supratherapeutic INR are paramount in preventing further complications, as demonstrated in our case. Administration of phytonadione (vitamin K_1_) and fresh frozen plasma efficiently restored therapeutic INR levels, leading to a remarkable improvement in the patient’s symptoms with conservative management. As many patients are routinely placed on oral anticoagulation, a heightened awareness among clinicians regarding this unique presentation is essential to avoid misclassification and mistreatment. The potential misdiagnosis of Ludwig angina in such cases could lead to unnecessary antibiotic use and subsequent delay in appropriate interventions. Fluctuations in INR levels, whether due to changes in diet, medication dosages, or other factors, can significantly impact the risk of bleeding complications. In raising awareness of pseudo-Ludwig angina, we aim to improve patient care, minimize the overuse of antibiotics, and facilitate the timely involvement of appropriate medical services. This case highlights the significance of clinical acumen, thorough examination, and prompt intervention in managing pseudo-Ludwig angina, ultimately enhancing patient outcomes in this distinctive clinical scenario.
